# Overexpression of PKMYT1 associated with poor prognosis and immune infiltration may serve as a target in triple-negative breast cancer

**DOI:** 10.3389/fonc.2022.1002186

**Published:** 2023-01-30

**Authors:** Huihui Li, Li Wang, Wei Zhang, Youting Dong, Yefeng Cai, Xiaoli Huang, Xubin Dong

**Affiliations:** ^1^ Department of Breast Surgery, The First Affiliated Hospital of Wenzhou Medical University, Wenzhou, China; ^2^ Department of Gastroenterology, Wenzhou Central Hospital, Wenzhou, China; ^3^ Shanghai Medical College, Fudan University, Shanghai, China; ^4^ Department of Thyroid Surgery, The First Affiliated Hospital of Wenzhou Medical University, Wenzhou, China

**Keywords:** PKMYT1, prognosis, multi-omics, TNBC subtypes, immune infiltration

## Abstract

Breast cancer (BC) is one of the most common malignancies among women worldwide. It is necessary to search for improvement in diagnosis and treatment methods to improve the prognosis. Protein kinase, membrane associated tyrosine/threonine 1 (PKMYT1), a member of the Wee family of protein kinases, has been studied in some tumors except BC. This study has explored that PKMYT1 functional role by bioinformatics methods combined with local clinical samples and experiments. Comprehensive analysis showed that PKMYT1 expression was higher in BC tissues, especially in advanced patients than that in normal breast tissues. The expression of PKMYT1 was an independent determinant for BC patients’ prognosis when combined with the clinical features. In addition, based on multi-omics analysis, we found that the PKMYT1 expression was closely relevant to several oncogenic or tumor suppressor gene variants. The analysis of single-cell sequencing indicated that PKMYT1 expression was upregulated in triple-negative breast cancer (TNBC), consistent with the results of bulk RNA-sequencing. High PKMYT1 expression was correlated with a poor prognosis. Functional enrichment analysis revealed that PKMYT1 expression was associated with cell cycle-related, DNA replication-related, and cancer-related pathways. Further research revealed that PKMYT1 expression was linked to immune cell infiltration in the tumor microenvironment. Additionally, loss-of-function experiments *in vitro* were performed to investigate the role of PKMYT1. TNBC cell lines’ proliferation, migration, and invasion were inhibited when PKMYT1 expression was knock-down. Besides, the down-regulation of PKMYT1 induced apoptosis *in vitro*. As a result, PKMYT1 might be a biomarker for prognosis and a therapeutic target for TNBC.

## Introduction

Breast cancer (BC) is the main cause of cancer-associated death among women across the world (1–3). According to statistics, 290,000 new breast cancer patients will be diagnosed in the United States in 2022 with over 40000 death cases (4). In the United States, it has been classified as the second most frequent cause of cancer mortality among women, leading to 6.9% of all cancer mortality (5). According to the latest Cancer statistics report in China, BC is also the most common malignant tumor among women in China, with an incidence of 29.05/100,000 and more than 70,000 people died of this disease in 2016, making it the fourth most common cancer death among women in China. Despite advances in the adoption of therapeutic strategies including improved surgical procedures and new targeted or immunotherapy drugs, the prognosis of BC is often still poor.

There is considerable heterogeneity in clinicopathologic features, prognostic outcomes, and genetic changes of BC patients. According to the expression of estrogen receptor (ER), progesterone receptor (PR), human epidermal growth factor receptor (HER2), and other indicators in patients, BC could be classified into various molecular subtypes, and corresponding treatment can be given to each subtype. In recent years, the prognosis of early BC has been significantly improved due to the progress of treatment methods, and the 5-year survival rate is over 85% (6). For example, CDK4/6 (cyclin-dependent kinase 4/6) inhibitors and HER2-targeted drugs (Pertuzumab, Trastuzumab, etc.) have been shown to reduce the risk of recurrence and prolong the survival of patients with early BC (7, 8). However, the 5-year survival rate for individuals with advanced BC is less than 40%, mainly due to continuous tumor invasion, metastasis, evolution, tumor resistance, and subsequent spread the malignancy throughout the body, leading to patient death (5, 9).

TNBC acts as a special type of breast cancer that is negative for ER, PR, and HER2, accounting for about 12-17% of all BC (10). Our previous retrospective analysis for 929 BC patients has shown that compared with other subtypes of breast cancer, TNBC patients are characterized by strong invasiveness, no targeted drug therapy, hormone insensitivity, poor prognosis, young-onset age, high recurrence and metastasis rate, high histological grade and later stage (11, 12). In recent years, researchers around the world have reclassified TNBC according to epigenetic level, DNA variation, transcriptome level, and other omics. Lehmann BD et al. have divided TNBC into six subtypes: basal-like 1 (BL1), basal-like 2 (BL2), immunomodulatory (IM), luminal androgen receptor (LAR), mesenchymal (M), and mesenchymal stem-like (MSL) (13), they also found different subtypes have different sensitivity to chemotherapeutic agents. This study provides an important theoretical basis for fully understanding the heterogeneity and precision treatment of TNBC. Similarly, Burstein et al. have proposed four molecular classifications for TNBC, including LAR, Mesenchymal (MES), Basal-Like immune-suppressed (BLIS), and Basal-Like Immune-Activated (14). Prognostic analysis showed that different subtypes have different tumor-free survival, with BLIA the best, MES the second-best, LAR slightly worse, and BLIS the worst. Based on the cohort of Chinese patients, Shao et al. also proposed the Fudan classification of TNBC, including LAR, IM, BLIS, and MSL. They proposed potential therapeutic targets based on the molecular characteristics of each subtype, such as immune checkpoint inhibitors, STAT3 inhibitors, and tumor stem cell inhibition (15). Therefore, it is urgent to find new key genes controlling the progression of TNBC, understand the mechanism of TNBC progression, and clarify the progression-related molecular targets.

Protein Kinase, Membrane Associated Tyrosine/Threonine 1 (PKMYT1), a gene belonging to the Wee1 G2 checkpoint kinase family, controls negatively the cell cycle (16). It serves as the kinase of MYT1 that efficiently phosphorylates CKD1/cyclin B complex in both threonine-14 (Thr14) and tyrosine-15 (Thr15) and prevents cells from transforming from G2 to the mitosis phase through two different pathways (17). One mechanism is that cytoplasmic PKMYT1 binds to the CKD1/cyclin B complex and inhibits the complex from entering the nucleus. PKMYT1 inhibits the activated CKD1 by phosphorylating Thr14/Thr15 residues on CKD1 (18). Previous studies suggest that Wee1 activity is crucial for maintaining the G2/M phase DNA damage checkpoints, exhibiting functional redundancy with PKMYT1 in CDK1 inhibition (17). The loss of *MYT1* (in the presence of Wee1) neither affects the timing of mitosis nor abrogates DNA damage checkpoints. However, a more recent study has shown that *MYT1* is essential for cell survival. At the same time, *PKMYT1* has been researched in the tumor tissues and found to be associated with oncogenic properties in several cancers, such as esophageal squamous cell carcinoma, lung adenocarcinoma, clear renal cell carcinoma, and prostate cancers (19–22); However, its diagnostic value and biological function have not been studied in BC. As the newest treatment for BC, immunotherapy is complementary to surgery, chemotherapy, hormone therapy, and targeted therapies. Immune checkpoint inhibitors like Pembrolizumab (anti-PD-1 antibody) have been proven to prolong the overall survival (OS) of advanced BC. However, there is considerable variation in the efficacy of immunotherapy due to the differences in TME. Of these, tumor-infiltrating immune cells influence the response and resistance to immune checkpoint blockade therapy, resulting in different prognostic outcomes for patients. Therefore, the evaluation of immune properties can predict the prognosis of patients with BC.

Based on the above findings, we identified PKMYT1 as a prognostic biomarker and explored the immune-related mechanism and functional roles of PKMYT1 in TNBC pathogenesis. Firstly, we explored the expression of PKMYT1 and its relationship to clinicopathological features of BC patients according to public datasets. Furthermore, we analyzed the correlation between PKMYT1 expression and prognosis outcomes of patients in BC. Based on the multi-omics analysis, we found that the expression of PKMYT1 was associated with several oncogenic or tumor suppressor gene variants. Furthermore, we found that PKMYT1 expression was higher in TNBC, especially in BL1 or BLIS, combined with multiple TNBC molecular subtypes. We identified the relationship between the PKMYT1 level and cancer-associated immune cells. Finally, we performed loss-of-function assays to explore the function of PKMYT1 in BC cell lines. Our study revealed that PKMYT1 was a prognostic biomarker with good diagnostic value and promoted TNBC cell growth.

## Materials and methods

### Data collection and processing

BC microarray data of the GPL570 platform from the GEO database was obtained from GENT2 (http://gent2.appex.kr/gent2/). Transcriptome sequencing data, single-cell sequencing data, and corresponding clinicopathologic data from GSE96058 and GSE75688 databases were obtained from the GEO database (https://www.ncbi.nlm.nih.gov/geo/). TCGA-BRCA cohort transcriptome sequencing data, DNA methylation data, copy number variation data, somatic gene mutation data, and clinicopathologic data were obtained from GDC Data Portal (https://portal.gdc.cancer.gov/). TCGA-BRCA transcriptome sequencing data were normalized with RSEM expression level per million transcripts. Microarray expression data, somatic gene mutation data, and clinicopathological data of the METABRIC dataset were obtained from cBioPortal (http://www.cbioportal.org/datasets). Transcriptome sequencing data, somatic gene mutation data, and clinicopathologic data from the FUSCCTNBC dataset were obtained from https://www.biosino.org (Project code OEP000155). Subtype classification data of TCGA and METABRIC cohorts of TNBC were obtained from previous studies (15, 23). We analyzed PKMYT1 mRNA expression in BC and normal samples using the breast GPL570 microarray data, TCGA-BRCA data, and local hospital BC surgical samples. The analysis for correlation between PKMYT1 mRNA expression and PKMYT1 copy number variation or methylation level was performed. The disease outcome of overall survival (OS) and recurrence-free interval (RFS) was analyzed by Kaplan–Meier (KM) method and log-rank tests using the “survival” package in the KM-plotter dataset (https://kmplot.com/analysis/) or METABRIC cohort. Cancer patients with complete clinical profiles were selected for the Cox regression analysis. Univariable and multivariable cox regression analyses were performed using the coxph function in the “survival” package. The analysis for PKMYT1 mRNA levels in different clinicopathologic levels, hub gene mutation (*TP53*, *PIK3CA*, and *MAP3K1*), and molecular subtypes were performed. Single-cell sequencing data from GSE75688 was analyzed. The raw gene expression matrix was generated and analyzed with the “seurat” package. The matrix was filtered by removing cell barcodes with < 200 expressed genes, > 6000 expressed genes or > 25% of reads mapping to mitochondrial RNA. The gene expression matrix was normalized and a quantile-normalized variance > 0.5 was selected as variable genes. All variably-expressed genes were used to construct principal components (PCs) and PCs covering the highest variance in the dataset were selected. Clusters were calculated with a resolution between 0.2-2 and visualized using the uMAP dimensional reduction method. Four types of cells including ER+HER2+, ER+HER2-, HER2+ and TNBC cells were defined. The R function “cor.test” used the Spearman method to evaluate the correlation analysis. We obtained genes with highly ranked PKMYT1 positive or negative correlation coefficients (Spearman correlation value > 0.5 or < - 0.5, *p* < 0.001) which were used for the heatmap.

Gene Ontology (GO), Kyoto Encyclopedia of Genes and Genomes (KEGG), and Gene set enrichment analysis (GSEA) analysis were performed for eligible genes, respectively. The gene set for GSEA was from the Molecular Signature Database (http://www.gsea-msigdb.org/gsea/msigdb). The abundance of cell types from the RNA-seq matrix were estimated using tumor microenvironment analysis. To examine the role of PKMYT1 expression on 22 different types of immune cells, transcriptome data in the TCGA-BRCA cohort was subjected to CIBERSORT analysis. *p* < 0.05 was considered statistically significant by the Pearson coefficient test and Wilcoxon rank sum test. The correlation analysis between PKMYT1 expression and drug sensitivity was carried out using the CellMiner database (http://discover.nci.nih.gov/cellminer/) (24, 25). Furthermore, we validated Tumor Immune Dysfunction and Exclusion (TIDE; http://tide.dfci.harvard.edu/) performance in predicting anti-PD1 and anti-CTLA4 response (26).

### Patients and breast tissues samples

BC and paired normal tissues were obtained from the Department of Breast Surgery, The First Affiliated Hospital of Wenzhou Medical University. Collected fresh tissues were immediately snap-frozen in liquid nitrogen and stored at -80°C for further RNA was detected.

### Cell cultures and RNA interference

The Shanghai Cell Resource Center provided the human BC cell lines and normal breast cells including MCF-10A, MDA-MB-231, BT-549, MCF-7, and BT-474. Cells were cultivated at 37°C in a 5% CO2 atmosphere with DMEM medium containing 10% fetal bovine serum (Gibca, USA). MDA-MB-231 and BT-549 cells were transfected with siRNA using Lipofectamine 3000 (CA, USA) in a 6-well plate. The sequence of siRNA targeting PKMYT1 was as follows: sense, 5’- GGACAGCAGCGGAUGUGUUTT-3’, antisense, 5’- GCGGUAAAGCGUUCCAUGUTT-3’.

### Quantitative real-time PCR

For quantitative real-time PCR (qRT-PCR), total RNA from cells was reverse-transcribed using PrimerScript reverse transcriptase (Toyobo, Japan). The data were analyzed by 2^−ΔΔCT^. The primers sequences (Sangon Biotech, Shanghai, China) were as follows: PKMYT1 forward primer, 5’-AGCAGCGGATGTGTTCAGTC-3’; PKMYT1 reverse primer, 5’-CAGAACGCAGCTCGGAAGAC-3’; GAPDH forward primer, 5’-CCATTTGCAGTGGCAAAG-3’; GAPDH reverse primer, 5’- CACCCCATTTGATGTTAGTG-3’.

### Cell proliferation assay

Cell counting kit-8 (CCK-8; Solaribo, China) assay was employed for measuring the proliferation of cells. MDA-MB-231 and BT-549 cells were seeded in 96-well plates with 100 ul medium supplemented with 10% FBS. After incubation for a specific time, cell proliferation was assessed. In 96-well plates, 10 μL of CCK-8 solution was added into individual wells. The cells were incubated for 3 hours at 37°C and the optical density was recorded at a wavelength of 450 nm.

### Colony formation assay

For the colony formation assay, 2000 cells were added to a 6-well plate and cultured for 2 weeks at 37°C with 5% CO_2_ atmosphere. After 2 weeks, cell colonies were washed with PBS. Cell colonies were stained with 0.5 percent crystal violet after being fixed in paraformaldehyde. The cell colonies were subsequently counted.

### Cell migration and invasion assay

In the migration experiment, MDA-MB-231 and BT-549 cells were seeded into the upper chamber. Cells possessing the capability of migrating could pass through the pore into the lower chamber with 10% serum. Non-migrated cells on the upper chamber were gently removed using a cotton swab after 24 hours of incubation. Methanol was used to fix cells migrating through the filter chamber, and 0.1% crystal violet was used to dye them. In the invasion experiment, the matrigel used to spread over the upper chamber. Other experimental procedures were the same as that in the migration assay. Finally, migrated or invaded cells were observed in randomly chosen fields with a 100× magnification microscope.

### Apoptosis assay

The Annexin V-FITC/PI apoptosis assay kit was used to detect apoptosis according to the manufacturer’s procedure (BD Biosciences, USA). Cells were washed in PBS before being treated with Annexin V-FITC and PI at 37°C in the dark for apoptosis detection (BD biosciences, USA). The flow cytometric analysis was performed for detecting cell apoptosis, and the data were analyzed using Flowjo 10 software (Tree Star Software, USA). The percentage of Q2 + Q3 was used to calculate the apoptosis rate.

### Statistical analysis

In the two-group analysis, the Mann–Whitney U test or the Student’s t-test was performed. The Kruskal–Wallis one-way analysis of variance was used to compare multiple groups. Spearman’s rank correlation was used to estimate correlation. Survival rates were compared using the log-rank test, and hazard ratios (HR) were calculated by the Cox proportional hazards model. For data analysis, R 4.3 and Graphpad Prism 8.1 were used.

## Results

### PKMYT1 was highly expressed in BC and could serve as an independent prognostic marker

**Figure 1:**
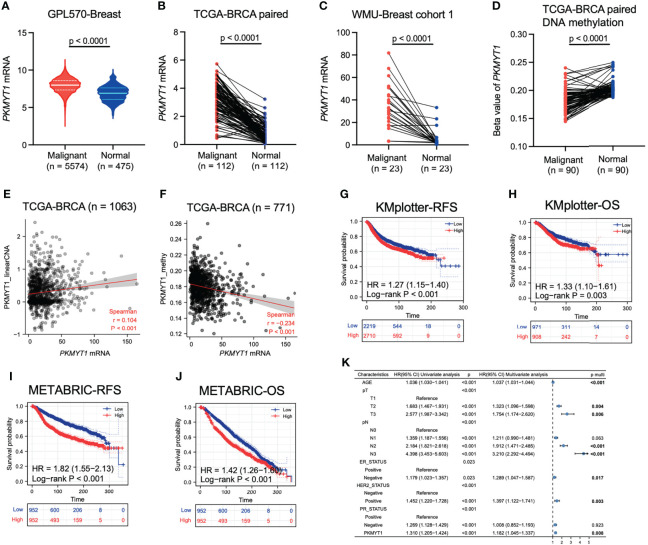
Analysis of multi-omics data showed that PKMYT1 was over-expressed in BC and could serve as an independent prognostic marker. **(A, B)** The PKMYT1 mRNA expression levels in BC microarray data and paired TCGA-BRCA cohort; **(C)** The mRNA expression levels of PKMYT1 in local cohort sample; **(D)** DNA methylation levels of PKMYT1 between BC and normal tissues were analyzed using HM450 chip sequencing data of TCGA-BRCA cohort; **(E, F)** The correlation between PKMYT1 mRNA expression level and PKMYT1 copy number variation or methylation level in TCGA-BRCA cohort; **(G–J)** Kaplan-Meier analysis for RFS and OS of BC patients in the KM-plotter cohort and METABRIC cohort; **(K)** Univariate and multivariate Cox regression analysis for OS of BC patients in METABRIC cohort.

The mRNA expression level of PKMYT1 in the integrated breast cancer GPL570 microarray data and the paired TCGA-BRCA cohort was significantly higher in breast cancer than that in the corresponding normal breast tissue, and this result was also verified in the small sample qRT-PCR cohort from our hospital (**Figures 1A–C**, *p* < 0.0001). Multiple omics data also confirmed that The DNA methylation level of PKMYT1 in breast cancer was lower than that in matched normal tissues (**Figure 1D**, *p* < 0.0001), and the mRNA level of PKMYT1 was positively correlated with the copy number variation (CNV) level of PKMYT1 (**Figure 1E**, *p* < 0.0001), while negatively correlated with methylation level (**Figure 1F**, *p* < 0.0001), indicating the potential association of PKMYT1 transcriptome level with genome level. In addition, the results of KMplotter and METABRIC data analysis also suggested that PKMYT1 high expression was associated with poor RFS and OS (**Figures 1G–J**, *p* < 0.001). Multivariate Cox regression analysis of the METABRIC cohort showed that old age, advanced T stage, advanced N stage, ER negative status, HER2 positive status, and PKMYT1 high expression were independent predictors for overall survival in BC (**Figure 1K**).

### PKMYT1 expression in BC was associated with clinicopathologic features and up-regulated in TNBC

**Figure 2:**
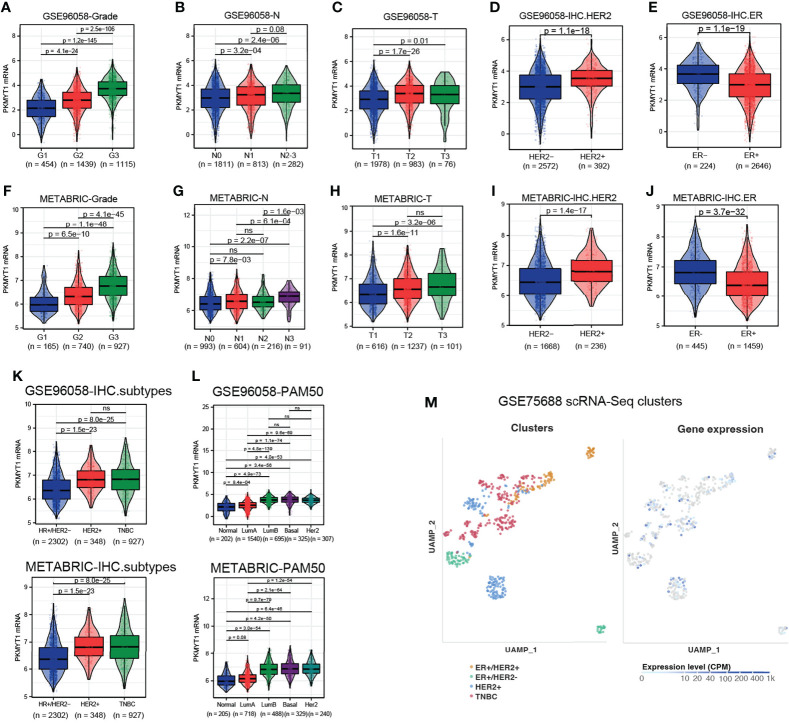
The correlation between PKMYT1 expression and clinicopathologic features in BC was analyzed using multi-source datasets. **(A–E)** The PKMYT1 mRNA expression levels in different tumor grade, tumor size stage, lymph node metastasis stage, HER2 status and ER status were analyzed in GSE96058 cohort. **(F–J)** The PKMYT1 mRNA expression levels in different tumor grade, tumor size stage, lymph node metastasis stage, HER2 status and ER status were analyzed in METABRIC cohort. **(K, L)** The PKMYT1 mRNA expression levels in different immunohistochemical molecular type or PAM50 molecular type were analyzed in GSE96058 and METABRIC cohorts. **(M)** Single-cell sequencing data analysis of GSE75688 cohort for PKMYT1 expression in different molecular types of breast cancer. ns, no significance.

A retrospective analysis of large sample data from GSE96058 and METABRIC cohorts showed that PKMYT1 expression was higher in high tumor grade, advanced tumor size stage, advanced lymph node metastasis stage, HER2 positive status, and ER negative status (**Figures 2A–J**). To further explore the expression level of PKMYT1 in different molecular types of BC, we found that the PKMYT1 level in TNBC and HER2-positive BC was significantly higher than that in hormone receptor-positive BC (**Figures 2K, L**). This result was verified in the single-cell sequencing dataset (GSE75688, **Figure 2M**).

### Co-expressed genes of PKMYT1 and predicted functions

**Figure 3:**
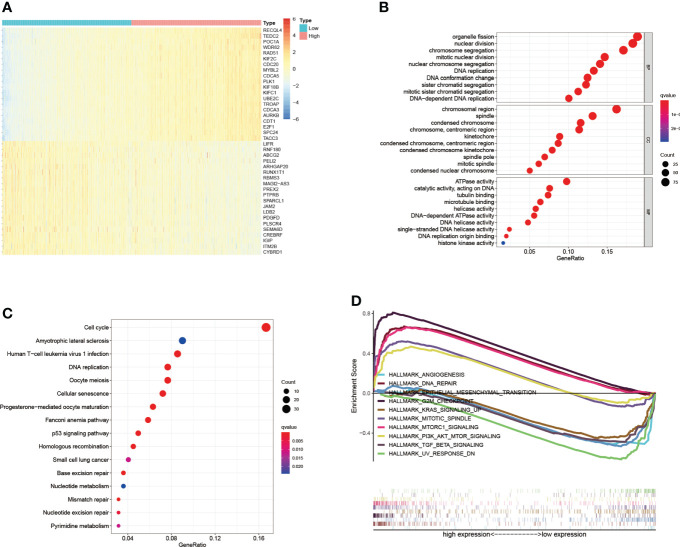
Co-expressed genes of PKMYT1 and function enrichment analysis. **(A)** The heatmap showed the top 40 genes strongly correlated with PKMYT1. **(B, C)** GO enrichment and KEGG enrichment analysis of genes co-expressed with PKMYT1. **(D)** GSEA analysis showed significant enrichments related to PKMYT1 expression.

Co-expression analysis of gene expression signatures helped understand underlying functions and pathways (27–29). To further explore the function of PKMYT1, co-expression analysis in the TCGA-BRCA cohort was performed to select genes that satisfied both of the following criteria: Spearman correlation value > 0.5 or < -0.5, and *p* < 0.001. The heatmap showed the top 40 genes positively and negatively correlated with PKMYT1 between high- and low-expressed groups (**Figure 3A**). To determine the function and pathway of these co-expressed genes, we performed GO and KEGG pathway enrichment analysis. GO analysis results including the biological process, cellular component, and molecular function showed that co-expressed genes were mainly enriched in organelle fission, nuclear division, DNA replication, chromosomal region, spindle, and ATPase activity (**Figure 3B**). Similarly, KEGG pathway enrichment analysis results showed the significant enrichment of genes in the cell cycle, DNA replication, amyotrophic lateral sclerosis, and p53 signaling pathway (**Figure 3C**). To further explore the functions of PKMYT1 in BC, we used hallmark gene sets to perform GSEA based on PKMYT1 expression. The results showed the expression of PKMYT1 was associated with angiogenesis, DNA repair, epithelial-mesenchymal transition, G2M checkpoint, Kras signaling, mitotic spindle, and mTORC1 signaling, PI3K-AKT-mTOR signaling, and TGF-beta signaling (**Figure 3D**).

### The expression of PKMYT1 correlated with the infiltrating immune cells

**Figure 4:**
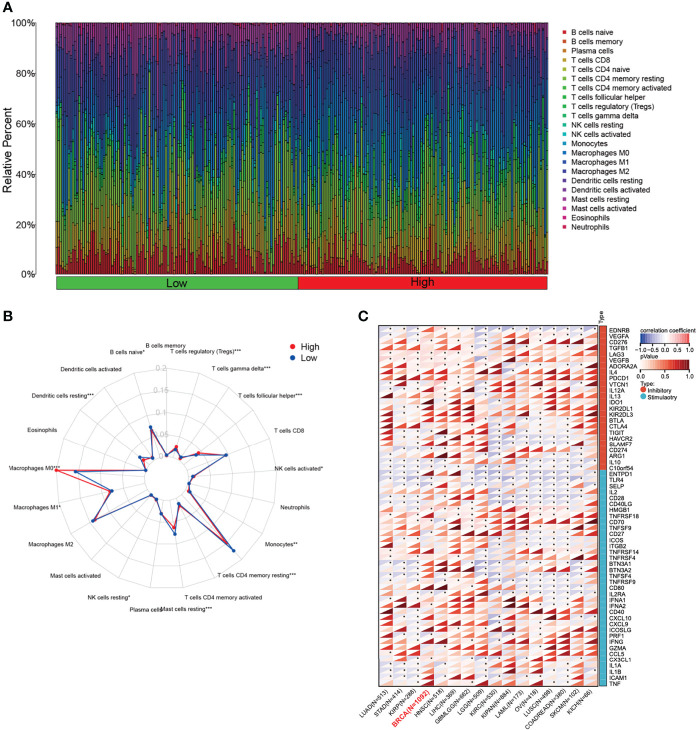
Correlation between the expression of PKMYT1 and immune infiltration. **(A)** The bar chart represented the component of immune cells in the high- and low-expressed PKMYT1 groups. **(B)** Comparison of different TME components between high and low PKMYT1 expression group. **(C)** Correlation between PKMYT1 and immune checkpoint mRNA level in various cancer type was analyzed in TCGA pan-cancer data. *p < 0.05, **p < 0.01, ***p < 0.001.

The changes in the immune microenvironment might affect the therapeutic effect of immunotherapy. CIBERSORT analysis was performed to estimate the proportions of tumor-infiltrating immune cells in BC (**Figure 4A**). The results showed that the proportions of naïve B cells, monocytes, memory resting T CD4 T cells, resting mast cells, and resting dendritic cells were lower in the high-expressed PKMYT1 group than those in the low-expressed group. Immunosuppressive cells like regulatory T cells and M0 macrophages are higher in the high-expressed PKMYT1 group (**Figure 4B**). We also found PKMYT1 was negatively correlated with CD274 mRNA level in TCGA-BRCA cohort (**Figure 4C**).

### PKMYT1 and drug response

**Figure 5:**
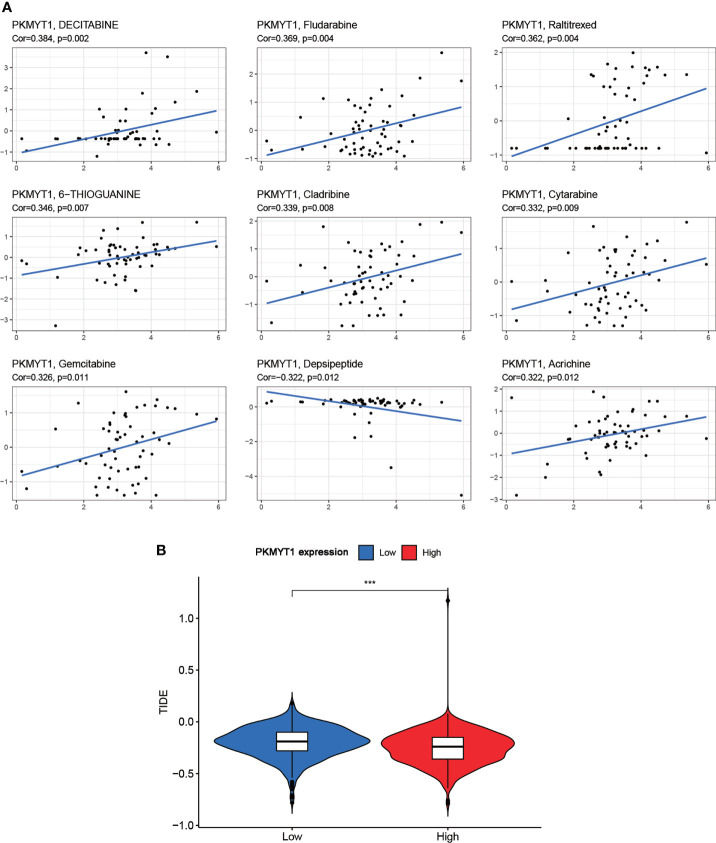
Correlation of PKMYT1 expression with the sensitivity of anticancer drugs and immunotherapy benefits. **(A)** The PKMYT1 expression was associated with drug sensitivity of Decitabine, Fludarabine, Raltitrexed, 6-Thioguanine, Cladribine, Cytarabine, Gemcitabine, and Depsipeptide. **(B)** TIDE between high and low PKMYT1 expression group. ***p < 0.001.

The drug sensitivity for PKMYT1 expression was performed to predict the sensitivity of BC patients to chemotherapy. PKMYT1 expression was positively associated with drug response in patients treated with Decitabine, Fludarabine, Raltitrexed, 6-Thioguanine, Cladribine, Cytarabine, Gemcitabine, and Acrichine (**Figure 5A**). Depsipeptide was negatively correlated with PKMYT1 expression. In addition, we validated TIDE performance in predicting anti-PD1 and anti-CTLA4 responses. The results showed that TIDE in the low-expressed PKMYT1 group was higher than that in the high-expressed group, indicating that patients in the high-expressed PKMYT1 group might be more sensitive to immunotherapy (**Figure 5B**).

### PKMYT1 was associated with a hotspot gene mutation in the genome that influence BC progression and with genomic molecular changes

**Figure 6:**
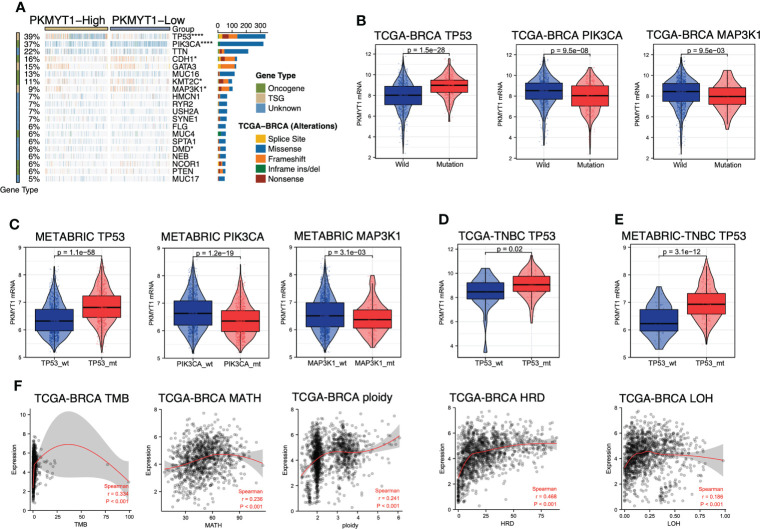
PKMYT1 was associated with key gene mutation and genomic molecular changes. **(A)** The waterfallmap from the TCGA-BRCA multi-omics cohort showed different mutant landscapes in high- and low-expression PKMYT1 groups; **(B, C)** PKMYT1 mRNA expression in TP53, PIK3CA and MAP3K1 mutation/wild-type group of TCGA-BRCA and METABRIC cohort; **(D, E)** PKMYT1 mRNA expression in the TP53 mutant/wild-type group of METABRIC and TCGA TNBC patients. **(F)** The Spearman correlation between PKMYT1 mRNA expression and genomic heterogeneity in TCGA cohort, including TMB (Tumor Mutation Burden), MATH (Mutant- Allele tumor heterogeneity), ploidy, HRD (Homologous recombination deficiency), and LOH (Loss of heterozygosity). *p < 0.05, ****p < 0.0001.

The mutant landscape between the high- and low-expression PKMYT1 groups was shown in the multi-omics dataset (**Figure 6A**). Mutations for tumor suppressor genes (*TP53* and *MAP3K1*) and oncogenes (*PIK3CA*, *CDH1*, and *KMT2C*) are significantly different in the high- and low-expression groups (**Figure 6A**). Next, we further explored that the PKMYT1 expression was up-regulated in BC somatic *TP53* mutant, *PIK3CA* wild-type, and *MAP3K1* wild-type groups (**Figure 6B**). Analysis in the METABRIC dataset also obtained similar results (**Figure 6C**). Interestingly, we found that the PKMYT1 expression was higher in the *TP53* mutant group of TNBC (**Figure 6D**), and the difference was more obvious in the METABRIC cohort (**Figure 6E**). Since *TP53* mutation is an important component of breast cancer genome heterogeneity, we analyzed the relationship between PKMYT1 expression and genome heterogeneity indicators. The results showed that PKMYT1 expression was correlated with TMB, MATH, ploidy, HRD, and LOH. (**Figure 6F**).

### PKMYT1 expression was highest in basal-like immune-suppressed TNBC

**Figure 7:**
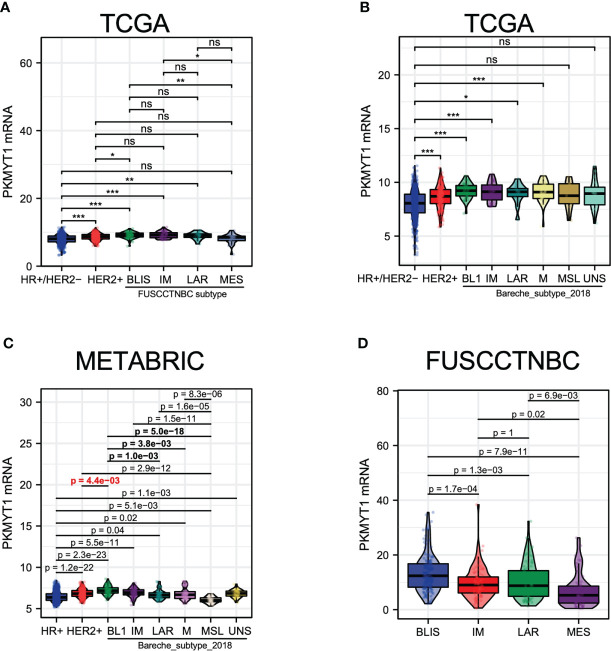
The PKMYT1 mRNA expression levels in different molecular types of TNBC. **(A)** The analysis for PKMYT1 expression in “Fudan classification “ and other molecular types of BC in the TCGA cohort. **(B)** The analysis for PKMYT1 expression in TNBC “Bareche classification” and other molecular types in the TCGA cohort. **(C)** METABRIC dataset analysis of PKMYT1 expression in TNBC “Bareche classification” and other molecular types. **(D)** The analysis for PKMYT1 expression in different TNBC molecular types in the FUSCCTNBC cohort. *p < 0.05, **p < 0.01, ***p < 0.001. ns, no significance.

Due to the high heterogeneity of TNBC, there was no significant difference in PKMYT1 expression between TNBC and HER2-positive BC (**Figures 2K, L**). To explore the correlation between PKMYT1 and TNBC, TNBC was further classified. We obtained and analyzed TNBC subtypes with large samples which were provided by different researchers worldwide. We obtained consistent results in different classification cohorts: Among all types of TNBC, PKMYT1 expression was highest in the basal-like immune-suppressed type, and PKMYT1 expression was higher in the basal-like immune-suppressed TNBC than that in the HER2-positive type in several cohorts (**Figures 7A–D**). Basal-like immune-suppressed TNBC was characterized by large tumor size, high proliferation activity of tumor cells, low density of immune-infiltrating cells in tumor mesenchyma, and high mutation frequency of *TP53*, *PTEN*, and *RB1*. Therefore, we verified the biological function of PKMYT1 in the TNBC cell.

### The expression of PKMYT1 was up-regulated in TNBC

**Figure 8:**
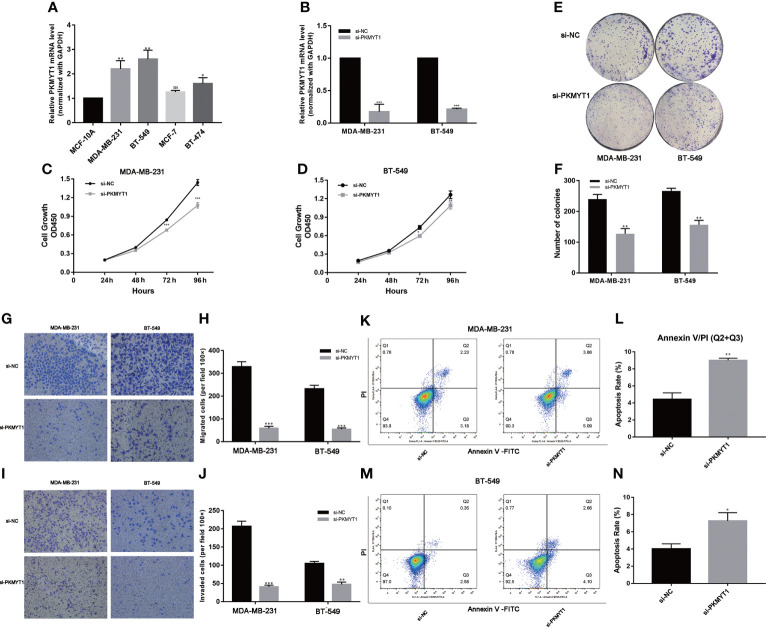
PKMYT1 promoted proliferation, colony formation, migration, invasion, and suppressed apoptosis in TNBC cell lines. **(A)** The qRT-PCR results showed PKMYT1 was up-regulated in TNBC cell lines. **(B)** The mRNA level of PKMYT1 in MDA-MB-231 and BT-549 TNBC cells transfected with si-NC and siPKMYT1. **(C, D)** Cell proliferation assay. **(E, F)** Colony formation assay. **(G–J)** Analysis of the effect of PKMYT1 on the migration and invasion of MDA-MB-231 and BT-549 cells. **(K, L)** The effect of PKMYT1 on MDA-MB-231 cells apoptosis by flow cytometry. **(M, N)** The effect of PKMYT1 on BT-549 cells apoptosis. *p < 0.05; **p < 0.01; ***p < 0.001. ns, no significance.

Based on these findings in public databases, we explored the biological function of PKMYT1 *in vitro* experiments. Then we found the mRNA expression of PKMYT1 was higher in BC cell lines (especially in TNBC cell lines, MDA-MB-231, and BT-549) than in non-tumorigenic cell lines MCF-10A (**Figure 8A**). MDA-MB-231 and BT-549 cells were for the loss-of-function experiments due to higher PKMYT1 expression. Then qRT-PCR results validated the efficiency of PKMYT1 knock-down by si-NC or si-PKMYT1 (**Figure 8B**).

### Down-regulation of PKMYT1 affected the colony-forming, proliferation, migration, invasion, and apoptosis of TNBC cells

The results of the above analysis suggested that PKMYT1 was up-regulated in BC, so we further explored its function in cell experiments. The results of CKK-8 and colony formation assays showed that down-regulated PKMYT1 could effectively inhibit BC cell proliferation and colony formation in MDA-MB-231, and BT-549 TNBC cell lines (**Figures 8C–F**). Transwell assays were used to investigate the effects of knockdown PKMYT1 on the invasion and migration capability in MDA-MB-231 and BT-549 cells. The results showed there were fewer cells migrated in the si-PKMYT1 group than in the si-NC group (**Figures 8G–J**), indicating that PKMYT1 knockdown could inhibit the migration and invasion capacities of TNBC cells. Further, we performed flow cytometry to detect apoptosis in MDA-MB-231 and BT-549 cells. The results showed that compared to TNBC cells in the si-NC group, those in the si-PKMYT1 group had significantly increased apoptotic ratio (**Figures 8K–N**).

## Discussion

BC is one of the most challenging health problems with high incidence and mortality (30, 31). The prognosis for BC is relatively poor and many patients are diagnosed at its advanced stage, especially in TNBC. There is a significant difference in treatment sensitivity in TNBC patients due to different tumor stages, tumor immune microenvironment and expression of driver oncogenes. Despite the emergence of many new treatments over the years including targeted therapy and immunotherapy, the prognosis of patients with advanced TNBC remains poor (32, 33). Therefore, it is urgent to find novel biomarkers for diagnosis, prognosis and targeted therapy. In this study, we focus on PKMYT1, a member of the WEE1 family, exerting a crucial effect on the assembly of Golgi apparatus and endoplasmic reticulum in mammalian cells. Recent studies have shown that PKMYT1 contributes to tumor progression *via* AKT/mTOR signaling pathway in esophageal squamous cell carcinoma (21). In renal clear cell carcinoma, radiation-induced G2/M phase arrest was eliminated when PKMYT1 was knocked down (19). Perez-Pea found five cycle-regulated genes which were associated with worse RFS and OS in breast cancer, including PKMYT1 (34). The researcher reported that PKMYT1 accelerates the malignant progression of ovarian cancer *via* negatively regulating SIRT3 (35). In lung adenocarcinoma, silencing PKMYT1 could prevent G2/M phase arrest and caused cells more sensitive to radiation (20). PKMYT1 promoted the growth of cells by targeting CCNB1 and CCNE1 in prostate cancer. In our study, we demonstrated for the first time that PKMYT1 was a prognostic marker according to public databases and promoted tumor progression with experiments in BC. Previous studies reported that PKMYT1 expression was up-regulated in many cancers. We analyzed the expression profile of PKMYT1 in BC and normal breast tissues. The results showed that the expression of PKMYT1 was up-regulated in BC tissues compared with normal or adjacent breast tissues, which was also verified in the small sample qRT-PCR cohort from our hospital. We found that the expression of PKMYT1 positively correlated with the CNV level, while negatively correlated with methylation level. Gene transcription levels would be regulated by many factors, including methylation, CNV, and alternative splicing. Usually, these factors interacted with each other. The study reported that independent CNVs could modulate gene expression and methylation, which in turn influenced each other (36). Further, we showed patients in the high-expressed PKMYT1 group had better outcomes in KM-plotter and METABRIC datasets. The univariate and multivariate Cox regression analyses for PKMYT1 and other clinicopathologic features were performed to indicate that PKMYT1 was an independent survival factor. Thus, our results showed that PKMYT1 expression was up-regulated in BC patients, and patients with higher PKMYT1 expression might have worse prognostic outcomes. Combined with clinicopathologic features, PKMYT1 was further over-expressed in advanced BC and TNBC.

Co-expression analysis was a common method of enhancing the generation of biologically relevant information and exploring biological functions. Furthermore, we carried out functional enrichment and co-expression analysis to assess PKMYT1’s biological roles. GO and KEGG enrichment analysis showed that PKMYT1 was mainly involved in organelle fission, nuclear division, DNA replication, chromosomal region, spindle, ATPase activity, cell cycle, amyotrophic lateral sclerosis, and p53 signaling pathway in previous studies. Tumor-associated signaling pathways like the p53 signaling pathway were also concerned. Besides, some cancer-related terms, such as Kras signaling, mTORC1 signaling, and PI3K-AKT signaling pathway, were enriched in the result of GSEA. Immunotherapy is the most recent BC treatment option and has the potential to become an important part of clinical cancer management (37–39), and TME participates in BC progression (38, 40). Our results indicate that immunosuppressive cells like regulatory T cells and M0 macrophages are associated with PKMYT1. It might cause an overall loss of tumor-associated antigen presentation, leading to immune resistance. According to our CellMiner database analysis, we found that the sensitivity of Decitabine was most associated with PKMYT1 expression, indicating that Decitabine might have the greatest antitumor efficacy for BC patients with high PKMYT1 expression. Based on the relationship between PKMYT1 and cancer-associated immune cells, we hypothesized that PKMYT1 could be connected with immunotherapy sensitivity. We found that patients in the high-expressed PKMYT1 group had lower TIDE, suggesting that might be more sensitive to immunotherapy. That suggested patients in the high PKMYT1 expression group were more suitable for immunotherapy.

TNBC was a highly heterogeneous subtype with strong invasiveness that had no opportunity for targeted therapy and hormone therapy. TNBC patients with genomic molecular changes and some special molecular subtypes were more likely to benefit from immunotherapy. TMB was highly correlated with the efficacy of PD-1/PD-L1 inhibitors. MATH, ploidy, and LOH indicated the heterogeneity of the tumor. HRD could produce specific, quantifiable, and stable genomic changes. Clinical research had found a strong correlation between HRD status and platinum Chemotherapy method or PARP inhibitor sensitivity. Our study showed that PKMYT1 expression was positively correlated with those genomic molecular changes.

In the experiments, we demonstrated that PKMYT1 expression was up-regulated in TNBC cells. We explored PKMYT1’s contribution to the proliferation, migration, and invasion of TNBC cells. Furthermore, PKMYT1 knockdown promoted apoptosis in TNBC cells. The biological function of PKMYT1 in TNBC was similar to that of other cancers. In our study, we confirmed *in vitro* experiments that PKMYT1 was an oncogene for patients with TNBC.

## Conclusions

In conclusion, the study confirms that the PKMYT1 is overexpressed in BC patients and could be a valuable prognostic marker. In addition, PKMYT1 expression is related to the cell cycle and tumor microenvironment, suggesting that it can help guide the use of clinical antitumor drugs according to the database. Lastly, the experiments demonstrate that PKMYT1 promotes TNBC cell growth, migration, and invasion, and suppresses apoptosis. Therefore, PKMYT1 could serve as a prognostic biomarker and therapeutic target in TNBC.

## Data availability statement

The original contributions presented in the study are included in the article/**Supplementary Material**. Further inquiries can be directed to the corresponding authors.

## Ethics statement

All research protocols were approved and implemented through the ethical standards of the institutional review board of the First Affiliated Hospital of Wenzhou Medical University (Approval No. 2012-57). The patients/participants provided their written informed consent to participate in this study.

## Author contributions

XD designed the study. HL, LW, and YD analyzed the data and performed the experiments. WZ and YC prepared the figures and finished the manuscript. XH provided revised suggestions for the manuscript. All authors contributed to the article and approved the submitted version.
